# Analog electro-optical readout of SiPMs for compact, low power ToF PET/MRI

**DOI:** 10.1186/2197-7364-1-S1-A12

**Published:** 2014-07-29

**Authors:** Matthew F Bieniosek, Craig S Levin

**Affiliations:** Department of Radiology, Stanford University, Stanford, CA 94305 USA; Molecular Imaging Program at Stanford (MIPS), Stanford, CA 94305 USA; Department of Physics, Stanford University, Stanford, CA 94305 USA; Department of Electrical Engineering, Stanford University, Stanford, CA 94305 USA; Department of Bioengineering, Stanford University, Stanford, CA 94305 USA

The aim of this work is to demonstrate time of flight (ToF) performance from analog electro-optical transmission of SiPM-based PET detector signals. In electro-optical readout schemes, scintillation signals are converted to near-infrared light by a laser diode and transmitted out of the MRI bore with fiber-optics [1], greatly reducing the PET system's footprint, power consumption, and mutual interference with the MRI.

**Figure 1:**
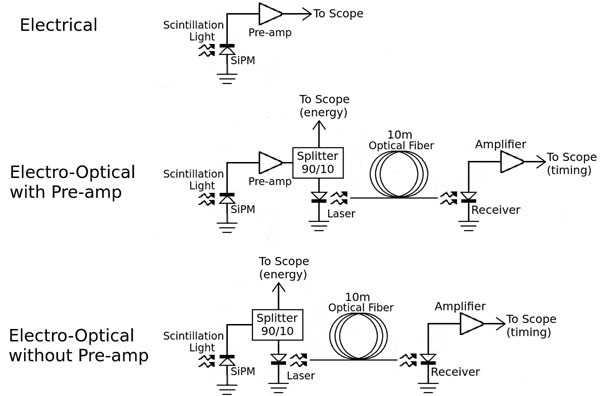
**Simplified schematics for the three data acquisition modes used in our experiments.** In electrical mode an amplified SiPM signal was sent directly to a high speed oscilloscope for timing pickoff. In electro-optical modes 10% of the analog SiPM signal (with or without amplification) was sent directly to the scope to for energy information. The other 90% was sent to the electro-optical link for timing pick-off.

**Figure 2:**
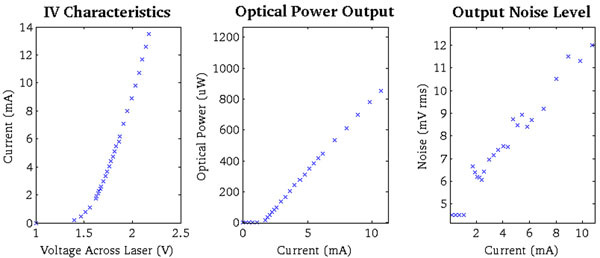
**Characterization of the HFE4192-58X VCSEL.** Its current rises exponentially with voltage (left). After the laser threshold current is surpassed, it's optical power (middle) and output noise levels (right) rise linearly with current. To optimize SNR a laser bias just above the VCSEL's threshold (1.7mA) was chosen.

Our approach uses vertical-cavity surface-emitting lasers (VCSEL) and _ber-optics borrowed from telecommunication systems to directly transmit analog signals from an SiPM (see Figure 1). Our experiments used 3mm x 3mm x 5 mm teflon wrapped, and 3mm x 3mm x 20mm ESR wrapped LYSO scintillation crystals, 3mm x 3mm C30742- 33-50-C SiPMs (Excelitas, Waltham, USA), ZX60-4016E+ preamplifiers (Minicircuits, Brooklyn, USA), HFE4192-58X VCSELs (Finisar, Sunnyvale,USA), and 10m multi- mode optical patch cables. The VCSEL was characterized as seen in Figure 2. To get the best analog performance a bias current just above the laser threshold (1.7 mA) was chosen to minimize noise, and power dissipation (2.8 mW).

**Figure 3:**
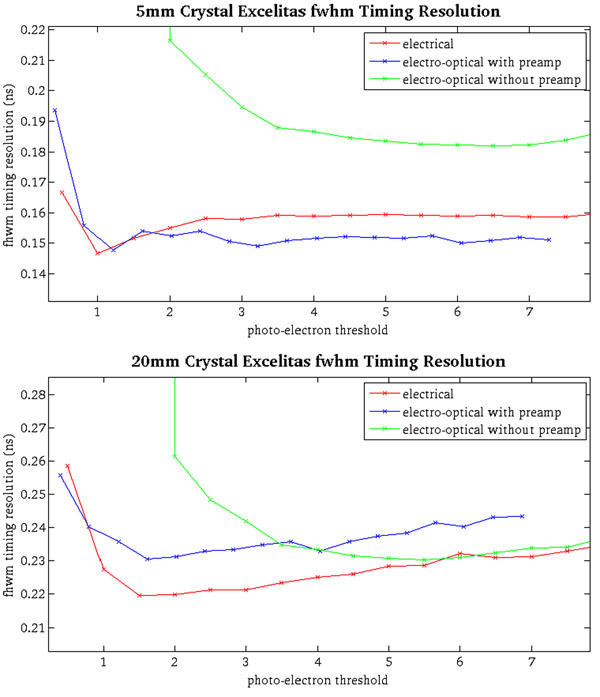
**Fwhm timing resolutions for each readout mode with 5mm length and 20mm length LYSO crystals at threshold levels normalized to the amplitude of a single photo-electron pulse for each readout mode.** The electro-optical readout degraded timing, but still maintained excellent performance.

The results of the timing experiments are seen in Figure 3. The best timing resolution achieved with 5mm length crystals was 147ps +/- 1ps in electrical mode, 148ps +/- 2ps in electro-optical with preamp mode, and 182 +/- 2ps in electro-optical without preamp mode. With 20mm length crystals the best timing achieved was 220 +/- 3ps in electrical mode, 230 +/- 2ps in electro-optical with preamp mode, and 230 +/- 2ps in electro-optical without preamp mode.

This work shows that SiPM ToF information can be preserved after analog electro- optical transmission. In the future this readout strategy could drastically simplify the design of high performance PET/MRI systems by reducing the in-bore electronics to two active components (SiPMs and VCSELs).
